# Cholesterol and hepatocellular carcinoma risk: reliable and actionable?

**DOI:** 10.1038/s41416-020-01249-x

**Published:** 2021-01-21

**Authors:** Alain Braillon

**Affiliations:** Previously senior consultant, 80000 Amiens, France

**Keywords:** Risk factors, Oncology

Yi and colleagues examined whether statin use was associated with a reduced risk of hepatocellular carcinoma (HCC) after consideration of cholesterol levels. Their conclusion “high cholesterol levels at statin initiation, not statin use, were associated with reduced risk of HCC” deserves to be questioned.

Firstly, their report was based on the data from participants of health examinations administered through the National Health Insurance Service (NHIS) of Korea in 2004–2007. It cannot be labelled as a “prospective cohort study”.^1^ The authors performed a post-hoc (retrospective) analysis of the observational data. Post-hoc analyses only generate a hypothesis and are in need of confirmatory studies. Have Yi et al. planned a confirmatory study?

Secondly, a PubMed search [“National Health Insurance Service” Korea] hit 182 publications. Sleight warned against post-hoc analyses by showing from the ISIS-2 trial that aspirin therapy was significantly different in patients born under the astrological signs of Gemini and Libra.^2^ Did Yi et al. pre-register their hypotheses (e.g., Open Science Framework) to avoid p-HARKing (hypotheses after the results are known)?

Thirdly, the reliability of the National Health Insurance Service cohort must be questioned. The annual incidence of HCC was 50/100,000 (1686 individuals with HCC among 400,318 people during an 8.4-years follow-up).^1^ This result contrasted with epidemiological studies from the Korean Liver Cancer Association: in Korea, the annual incidence of HCC continuously increased from 28.2/100,000 in 1999 to 32.7 in 2010 and then after have been plateauing at 31–32, up to 2015.^3^ What is the cause of such a large discrepancy? Could liver metastases wrongly be classified as HCC, a frequent flaw in administrative databases?^4^

Fourthly, most confounding variables were classified in arbitrarily categories despite being continuous with no modal distributions. For example, tobacco use was classified into three categories (current smoker, former smoker and never smoker). Such rough classifications preclude coherence of adjustments and complex statistical analyses cannot overcome the poor quality of the data. Further, tobacco is a dose-related (exponential) cause of HCC, while the analysis performed used a linear model which also failed to account for synergistic effects (e.g., tobacco and alcohol).

Tobacco ranks first among causes of HCC, far before hepatitis or alcohol, even in Asia.^5,6^ In the National Health Insurance Service cohort, 61% of those without HCC were never smokers vs only 49 of those HCC and only 45% of those with HCC were abstinent from alcohol vs 55 of those without HCC (Supplementary Table S1 in ref. ^1^). Could Yi et al. provide the attributable fractions of alcohol and tobacco? Such an estimate would give the proportional reduction in disease incidence that would occur if risk factor exposure were reduced to an alternative ideal exposure level (i.e., no tobacco use).

I also encourage the authors to adopt the STROBE checklists, an international epidemiological initiative aiming at “STrengthening the Reporting of OBservational studies in Epidemiology”. STROBE should be implemented to ensure scientific rigour.

## Data Availability

Not applicable.
